# Hepatocyte Aquaporins AQP8 and AQP9 Are Engaged in the Hepatic Lipid and Glucose Metabolism Modulating the Inflammatory and Redox State in Milk-Supplemented Rats

**DOI:** 10.3390/nu15163651

**Published:** 2023-08-20

**Authors:** Giovanna Trinchese, Patrizia Gena, Fabiano Cimmino, Gina Cavaliere, Chiara Fogliano, Sabino Garra, Angela Catapano, Lidia Petrella, Silvia Di Chio, Bice Avallone, Giuseppe Calamita, Maria Pina Mollica

**Affiliations:** 1 Department of Biology, University of Naples Federico II, 80126 Naples, Italy; giovanna.trinchese@unina.it (G.T.); fabiano.cimmino@unina.it (F.C.); chiara.fogliano@unina.it (C.F.); angela.catapano@unina.it (A.C.); lidia.petrella@unina.it (L.P.); bice.avallone@unina.it (B.A.); mariapia.mollica@unina.it (M.P.M.); 2 Department of Biosciences, Biotechnologies and Environment, University of Bari Aldo Moro, 70125 Bari, Italy; annapatrizia.gena@uniba.it (P.G.); sabino.garra@uniba.it (S.G.); 3 Department of Pharmaceutical Sciences, University of Perugia, 06126 Perugia, Italy; gina.cavaliere@unipg.it; 4 Azienda Sociosanitaria Territoriale Fatebenefratelli (ASST FBF) SACCO, University of Milan, 20157 Milan, Italy; silvia.dichio@asst-fbf-sacco.it; 5 Task Force on Microbiome Studies, University of Naples Federico II, 80138 Naples, Italy

**Keywords:** liver, aquaglyceroporins, peroxiporins, mitochondria, ROS

## Abstract

Milk is an important source of nutrients and energy, but there are still many uncertainties regarding the health effects of milk and dairy products consumption. Milk from different species varies in physicochemical and nutritional properties. We previously showed that dietary supplements with different milks in rats trigger significant differences in metabolic and inflammatory states, modulating mitochondrial functions in metabolically active organs such as the liver and skeletal muscle. Here, we have deepened the effects of isoenergetic supplementation of milk (82 kJ) from cow (CM), donkey (DM) or human (HM) on hepatic metabolism to understand the interlink between mitochondrial metabolic flexibility, lipid storage and redox state and to highlight the possible role of two hepatocyte aquaporins (AQPs) of metabolic relevance, AQP8 and AQP9, in this crosstalk. Compared with rats with no milk supplementation, DM- and HM-fed rats had reduced hepatic lipid content with enhanced mitochondrial function and decreased oxidative stress. A marked reduction in AQP8, a hydrogen peroxide channel, was seen in the liver mitochondria of DM-fed rats compared with HM-fed, CM-fed and control animals. DM-fed or HM-fed rats also showed reduced hepatic inflammatory markers and less collagen and Kupffer cells. CM-fed rats showed higher hepatic fat content and increased AQP9 and glycerol permeability. A role of liver AQP8 and AQP9 is suggested in the different metabolic profiles resulting from milk supplementation.

## 1. Introduction

Nutritional habits are undisputedly the main driver of metabolic homeostasis. Healthy nutrition, together with physical activity, has a positive influence on metabolic health and the immune system and lowers the risk of chronic and communicable diseases [1,2,3,4,5]. Appropriate dietary recommendations, helping to control body weight, glycemia, insulin sensitivity and lipid metabolism, are effective in limiting metabolic disorders and the occurrence of related chronic pathologies [6,7,8,9]. Indeed, countless studies demonstrated the impact of specific diets on the processes of inflammation, mitochondrial function and redox balance, tightly involved in the regulation of energy homeostasis [10,11,12,13,14,15]. However, we are still quite far from identifying precise molecular pathways activated in response to the intake of specific nutrients. For years, we have been studying the metabolic and non- effects of the consumption of a pivotal food in our diet. Milk is consumed by more than 6 billion people worldwide; not only it is a staple food for infant nutrition, but humans keep drinking milk after weaning, reinforcing its role as an important macro- and micronutrients source [16,17,18,19,20,21,22]. Our previous studies reported that the administration of milk of different species influences the metabolic and inflammatory profile of the recipient at different levels. It was demonstrated that donkey and human milk administration in rats increases energy expenditure, reduces body weight gain, body lipid depots and energy efficiency and ameliorates the inflammatory and oxidative state compared with cow milk administration [23,24]. In particular, our studies have shown that underlying this metabolic and inflammatory regulation are two striking conducting mechanisms: (i) the modulation of mitochondrial energy function and efficiency in metabolically active organs, such as the liver, skeletal muscle and heart [23,24,25], and (ii) the modulation of the composition and richness of gut microbiota [25,26].

In this framework, the liver is the main organ that coordinates the body’s metabolic flexibility, balancing immune and metabolic homeostasis [27,28,29,30]. The liver manages the flow of nutrients and endocrine and immune mediators, delivered via portal blood from gut and visceral adipose tissue, which modulate the hepatic metabolism influencing the bioenergetic regulation in hepatic mitochondria, organelles acting as a metabolic hub for the regulation of hepatocyte homeostasis [31,32,33]. In addition, the liver plays key metabolic functions. As the main target of insulin and glucagon signaling, the liver is pivotal in modulating glucose blood levels by regulating glycogen synthesis and gluconeogenesis. It is also a key organ in sustaining lipid homeostasis through fatty acid oxidation (taking place into mitochondria) and has the unique capacity to synthesize fatty acids by de novo lipogenesis and to secrete and take up lipoproteins [34,35].

Aquaporins (AQPs) represent a family of membrane channel proteins largely expressed in living organisms, where they play a series of important roles [36,37]. Humans possess 13 distinct homologues (AQP0-12), variously expressed in all body districts and roughly subdivided in two main groups based on their channel permeability, the *orthodox aquaporins,* AQPs allowing the movement of only water, and the *aquaglyceroporins*, AQPs that facilitate the movement of glycerol and other neutral solutes in addition to water. A subset of AQPs, the so-called *superaquaporins* (AQP11 and 12), has a distinct evolutionary pathway and has intracellular localization [38]. The AQPs also allowing permeation of ammonia are named *ammoniaporins*, while those that also facilitate the transmembrane movement of hydrogen peroxide are named *peroxiporins* [39]. AQP8 and AQP9 are by far the main AQPs expressed in hepatocytes, where they are considered to be of remarkable relevance [40,41]. AQP8 is an AQP permeable to ammonia, hydrogen peroxide and water. In hepatocytes, AQP8 has multiple subcellular localizations and has been suggested to mediate the secretion of canalicular bile water [42], to preserve the cytoplasm osmolarity during the synthesis and degradation of glycogen, to conduct ammonia in mitochondrial ammonium detoxification and ureagenesis [43,44] and to facilitate the release of hydrogen peroxide from mitochondria [45]. AQP9 is an aquaglyceroporin of broad selectivity allowing transmembrane movement of a wide variety of noncharged solutes, including glycerol and other polyols, hydrogen peroxide, urea, carbamides, nucleosides, monocarboxylates, purines, pyrimidines and metalloid arsenic, besides water [46]. Liver exerts a central function in glycerol metabolism, considering its predominant (70–90%) contribution to the whole-body glycerol metabolism [47,48]. In hepatocyte, AQP9 is localized at the sinusoidal plasma membrane [49], where it facilitates the import of glycerol from the portal blood during short-term fasting to make glucose through gluconeogenesis after its conversion into glycerol-3-phosphate (G3P) [50,51,52,53]. Involvement of AQP9 in lipid homeostasis was also reported in the hepatic synthesis of triacylglycerols (TAGs) [54]. Functional significance for AQP9 in glucose and lipid homeostasis and energy balance was also indicated by studies with *Aqp9* knockout mice, where the ablation of AQP9 led to reduced liver glycerol permeability and increased levels of plasma glycerol and TAGs [55]. Hepatocyte AQP9 has also been suggested to contribute to rodent bile formation and to the extrusion of catabolic urea [53,55]. In rodents, the transcriptional expression of hepatocyte AQP9 is negatively regulated by insulin [56]. Both murine models of obesity and patients with obesity and type 2 diabetes have reduced levels of hepatic AQP9 accompanied by a decrease in liver glycerol permeability [57,58]. Hepatocyte AQP9 is also found to be regulated by leptin [54]. However, the regulation played by both insulin and leptin on the gene transcription of AQP9 seems to differ between rodents and humans [53]. Hepatic AQP9 expression shows gender-specific dimorphism both in rodents and humans in line with the known differences with which the two sexes manage glycerol (see Rodríguez et al. (2015) [59] and Lebeck (2023) [60] for review).

The aim of this work was to investigate the effects of isoenergetic supplementation of milk (82 kJ) from cow (CM), donkey (DM) or human (HM) on hepatic metabolism to improve the knowledge of the coordinated interlink between glucose metabolism, lipid storage and redox state and to highlight the possible role of two hepatocyte aquaporins, AQP8 and AQP9, in this crosstalk.

## 2. Materials and Methods

### 2.1. Animals and Chemicals

All chemicals were purchased from Sigma–Aldrich (St. Louis, MO, USA), unless otherwise stated. Cow milk (CM) was obtained from “Nuova Latte Soc. Coop. Agr. A R.L.” (Contrada Isca SNC, Eboli, SA, Italy). Donkey milk (from the Ragusana breed) (DM) was obtained from “Az. Agric. Garofalo Patrizia” (Contrada Valle Cerasa, Casalbordino, Italy). Human milk (HM) was kindly provided by the milk bank of the Macedonio Melloni Hospital (Department of Childhood and Evolutionary Age Medicine, Milan, Italy). Standard rodent diet (4RF21) was purchased from Mucedola (Mucedola srl, Settimo Milanese, Italy). Male Wistar rats (60 days old; 345 ± 7 g; Charles River, Calco, Lecco, Italy) were caged in a temperature-controlled room and exposed to a daily light–dark cycle (12/12 h) with free access to chow diet (15.88 kJ/g) and drinking water. The rats were divided into four experimental groups (*n* = 7), three of them were supplemented with equicaloric intake (82 kJ) of raw CM, DM or HM, drinking 21, 48 or 22 mL/day, respectively. The milk was provided as a beverage through the use of feeding bottles. The animals were treated for 4 weeks. The last group did not receive milk supplement and was used as control. The diet composition is specified in Table 1. Despite the different volumes used, the metabolized energy by the different milk supplements was kept virtually the same (Table 2). The body weights of the animals were monitored three times a week (see Supplementary Table S1). At the end of the treatments, the animals were anesthetized by intraperitoneal injection of chloral hydrate (40 mg/100 g body weight), sacrificed by decapitation, and venous blood was taken from the inferior cava. Livers were removed quickly and fixed for 24 h in 4% buffered paraformaldehyde (PFA) and then embedded in paraffin for histochemical analysis or frozen using liquid nitrogen and stored at −80 °C. Animal experiments were carried out following the Directive 2010/63/UE, enforced by Italian D.L. 26/2014, and approved by the animal care and the Committee of the University of Naples “Federico II” (OPBA), Naples, Italy and the Italian Ministry of Health, Rome, Italy (authorization n. 97/2019-PR).

### 2.2. Oral Glucose Tolerance Test and Insulin Tolerance Test

For the glucose tolerance test, rats were fasted overnight to subsequently be orally dosed with glucose (3 g/kg body weight) dissolved in water. For the insulin tolerance test, mice were fasted for 5 h to normalize blood glucose and then challenged by an intraperitoneal injection of insulin (homolog-rapid-acting, 10 unit/kg body weight in sterile saline; Novartis, Basel, Switzerland). Blood was collected by direct flow from a small tail slit, before and after treatment at given intervals, and glucose levels were determined using a rat-calibrated glucose monitor (Contour XT, Ascensia Diabetes Care, Milan, Italy) and the insulin levels by ELISA (Mercodia rat insulin; Mercodia, Uppsala, Sweden). Basal fasting values of serum glucose and insulin were used to calculate Homoeostatic Model Assessment (HOMA) index as (Glucose (mg/dL) * Insulin (mU/L))/405 [61].

### 2.3. Hepatic Inflammatory and Oxidative State

Specific ELISA kits were used to measure tissue levels of interleukins (IL-1, IL-6 and IL-10; Thermo Scientific, Rockford, IL, USA) and tumor necrosis factor-α (TNF-α) (Biovendor R&D, Brno, Czech Republic). The rate of mitochondrial H_2_O_2_ release was assayed by following the linear increase in fluorescence (wavelength of excitation and emission of 312 and 420 nm, respectively) due to the oxidation of homovanillic acid in the presence of horseradish peroxidase [62]. Superoxide dismutase (SOD) specific activity was measured in a medium containing 0.1 mM EDTA, 2 mM KCN and 50 mM KH_2_PO_4_, pH 7.8 and after the addition of 20 mM cytochrome c, 5 mM xanthine and 0.01 U of xanthine oxidase. Enzyme activity was monitored spectrophotometrically (550 nm) at 25 °C by tracking the decline in the reduction rate of cytochrome c by superoxide radicals, generated by the xanthine–xanthine oxidase system. One unit of SOD activity is established as the concentration of enzyme that inhibits cytochrome c reduction by 50% in the presence of xanthine and xanthine oxidase [63]. Lipid peroxidation was detected in liver homogenates by measuring the levels of malondialdehyde (MDA) with the thiobarbituric acid (TBAR) method. The MDA content was determined using a colorimetric assay kit according to the manufacturer’s protocol (MAK085). Equal volumes (100 μL) of sodium dodecyl sulfate and sample were mixed in a 5 mL conical vial. The mixture was added to 0.4 mL of 1% thiobarbituric acid in 0.2 mL (20%) H_3_PO_4_ and 50 mM NaOH and was mixed gently. The mixture was incubated on ice before being heated for 15 min at 100 °C. The samples were then centrifuged for 10 min at 1600× *g* and, lastly, their absorbance was read at 540 nm. MDA values were expressed as micromoles per milligram of protein. Protein Carbonyl accumulation (PC), as an index of protein oxidative modification, was measured in the liver, as previously reported [64]. Catalase activity was determined as the decomposition of H_2_O_2_ at 25 °C [65]. The levels of ROS were determined by diluting an appropriate volume of freshly prepared tissue homogenate in 100 mM potassium phosphate buffer (pH 7.4) and incubated with a final concentration of 5 μM dichlorofluorescein diacetate in dimethyl sulfoxide for 15 min at 37 °C. The dye-loaded samples were centrifuged at 12,500× *g* for 10 min at 4 °C. The pellet was mixed at ice-cold temperature in 5 mL of 100 mM potassium phosphate buffer (pH 7.4) and again incubated for 60 min at 37 °C. The fluorescence measurements were obtained by using the HTS-7000 Plus plate reader spectrofluorometer (Perkin Elmer, Wellesley, MA, USA) at 488 nm and 525 nm for excitation and emission wavelengths, respectively. ROS were quantified from the dichlorofluorescein standard curve in dimethyl sulfoxide (0–1 mM) [66].

### 2.4. Semiquantitative Reverse Transcription PCR

Total RNA was extracted from the liver of sacrificed rats using Tri Reagent Solution (Ambion, Foster City, CA, USA) according to the manufacturer’s instructions. Two micrograms of total RNA extracted from each sample was reverse transcribed to cDNA using HighCapacity cDNA Reverse Transcription Kit (Applied Biosystems, Foster City, CA, USA). Equal amounts of the resulting cDNA were used as templates for the subsequent PCR reactions performed to analyze the transcriptional expression of *AQP8* and *AQP9* by using AmpliTaq DNA Polymerase (Applied Biosystems) and the primer pairs rAQP8start (5′-ATGTCTGGGGAG CAGACG-3′) and rAQP8stop (5′-CGCCTGATTCTAAAGTCGAGG-3′) and rAQP9start (5′-ATGCCTTCTGAGAAGGACGGT-3′) and rAQP9stop (5′-AGCTCAGTGTCATCATGTAG-3′), respectively. RT-PCR reactions were performed as previously reported [57] and internally normalized against the β-actin expression.

### 2.5. Isolation of Liver Mitochondria and Hepatocyte Sinusoidal Membrane Vesicles

Rat liver mitochondria and vesicles of hepatocyte sinusoidal plasma membrane were processed as previously reported [50]. Briefly, mice livers were quickly removed after the sacrifice and homogenized with a Potter–Elvehjem homogenizer (15 strokes at 500 rpm) in an ice-cold isolation medium (220 mM mannitol, 70 mmol/L sucrose, 20 mmol/L Tris-HCl, 1 mmol/L EDTA and 5 mmol/L EGTA; pH 7.4) and added to a cocktail of protease inhibitors (complete Protease Inhibitor Cocktail, Roche Italia, Monza, Italy). The homogenate was centrifuged at 500× *g* for 10 min, and the pellet consisting of nuclei and unbroken cells was discarded. The resulting supernatant was centrifuged at 8000× *g* for 20 min. The related pellet containing mainly mitochondria was collected (*mitochondrial fraction*), whereas the supernatant was centrifuged at 10,000× *g* for 15 min. The resulting pellet enriched in hepatocyte sinusoidal membrane vesicles was collected, and the protein concentration was assayed by BCA colorimetric approach (Pierce, Thermo Fisher Scientific, Rockford, IL, USA). All centrifugations were carried out at 4 °C. Enrichment and purity of mitochondria and isolated sinusoidal membranes were comparable to those previously reported [50,67].

### 2.6. Western Blot Analysis

Aliquots (10 μg of total proteins) of rat liver mitochondria prepared as described above were heated to 90 °C for 5 min and electrophoresed into 10% NuPAGE Bis-Tris precast polyacrylamide gels (Invitrogen, Waltham, MA, USA) using a SeeBlue™ Plus2 prestained protein ladder (Invitrogen). The resolved proteins were submitted to immunoblotting and incubated overnight with affinity-purified rabbit antibodies against an N-terminal peptide of rat AQP8 at a final concentration of 1 μg/mL blocking solution (20 mM Tris–HCl, 0.15 M NaCl, 1% Triton X-100, pH 7.5). Horseradish peroxidase antirabbit IgG-treated membranes (antirabbit IgG peroxidase antibody; Sigma) were developed by luminal chemiluminescence (ECL WEST FEMTO PLUS, Immunological Sciences, Rome, Italy). The immunoreactive bands were analyzed by densitometry using the ImageJ software (National Institutes of Health, Bethesda, MD, USA). The density of each band was normalized against that of the housekeeper gene β-actin.

### 2.7. Stopped-Flow Light Scattering Measurements of Glycerol Permeability

The size of the sinusoidal membrane vesicles prepared as above was determined using an N5 Submicron Particle Size Analyzer (Beckman Coulter, Palo Alto, CA, USA) and by transmission electron microscopy. The time course of vesicle volume change was followed from changes in scattered light intensity at 20 °C at the wavelength of 530 nm by using a BioLogic MPS-200 stopped-flow reaction analyzer (BioLogic, Claix, France) that has a 1.6 ms dead time and 99% mixing efficiency in <1 ms. For the glycerol permeability measurements, light scattering experiments were performed as previously described by submitting the vesicles to a 150 mmol/L inwardly directed gradient of glycerol [35]. The glycerol permeability coefficient (*P_gly_*; cm/s) was computed by using the following equation:

*P_gly_* = 1/[(S/V)τ]

where S/V is surface-to-volume ratio and τ is the exponential time constant fitted to the vesicle swelling phase of the light scattering time course corresponding to glycerol entry.

### 2.8. Histological Analyses

Following the sacrifice, a piece of liver from each animal (treated and control groups) was cut into 4 blocks of 4–5 mm^3^, belonging to different areas. Each block was immediately fixed in 4% PFA for 24 h at 4 °C. Samples were then processed for wax embedding according to [68], and serial sections (5 µm) were cut. Hematoxylin-eosin staining was used to highlight the general morphology; periodic acid–Schiff (PAS) was used to underline mucin, glycogen and glycoproteins [69]; and the alternative PAS/diastase was performed to highlight all the PAS-positive substances except for the glycogen, that is, digested by diastase, while PAS/dimedone (5,5-dimethyl-1,3-cyclohexanodione) was used to specifically identify the glycogen. Dimedone, in fact, digests all the other PAS-positive substances. Sections were oxidized in 0.5% periodic acid solution for 10 min (or first pretreated with diastase for 30 min), rinsed in double-distilled water and stained with Schiff’s reagent in the dark for 45 min (or first pretreated for 3 h at 60 °C with a 5% aqueous solution of dimedone). The reaction was blocked by repeated washing in 2.5% sodium bisulfite in 0.05 N HCl. Lastly, hematoxylin was utilized to nuclei contrast. Finally, Sirius Red staining was used to assess the presence of fibrosis. Briefly, liver sections were stained in Weigert hematoxylin solution for 8 min and placed in 0.1% Sirius Red solution dissolved in aqueous saturated picric acid for 1 h and dehydrated and mounted with DPX Mounting. All stains were performed on serial slides, and comparisons on the same liver areas were performed. Images were acquired with a Zeiss Axiocam camera applied to a Zeiss Axioskop microscope, and counting of Kupffer cells [70] was performed by selecting images at 40× magnification obtained by 50 sections derived from different areas and analyzed with AxioVision 4.7 Software (Zeiss, Oberkochen, Germany).

### 2.9. Statistical Analysis

All data were presented as means ± SEM. Differences among groups were compared by one-way ANOVA followed by Tukey’s post hoc test to correct for multiple comparisons. Data were analyzed using GraphPad Prism 6.0 (GraphPad Software Inc., La Jolla, CA, USA) with *p* ≤ 0.05 as the cut-off for statistical significance between groups. Data with different superscript letters were significantly different according to the statistical analysis, as specified in the figures and tables.

## 3. Results

### 3.1. Histological Analyses

**Figure 1:**
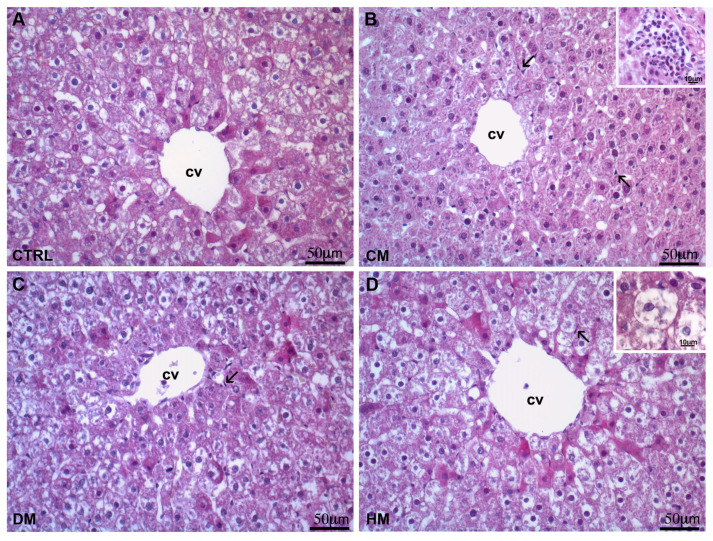
Hematoxylin-eosin staining: (**A**) Liver of control rats. As expected, the liver has the typical parenchymal architecture with cell hepatocyte cords starting from the centrilobular vein and divided by the hepatic sinusoids. (**B**) CM-fed rats liver displayed increased Kupffer cells (arrows) and inflammatory infiltrates (inset). (**C**) No differences are seen between the livers of DM-fed and control rats. (**D**) Hypertrophic hepatocytes (inset) are observed around the centrilobular vein of the liver of HM-fed rats. cv: centrilobular vein. The arrows indicate the Kupffer cells.

To start, histological analyses were run with samples of the different liver specimens to evaluate the effects of milk supplementations on the hepatic architecture, pattern of accumulation of glycogen and other polysaccharides, Kupffer cells proliferation and fibrosis. The results of the hematoxylin-eosin staining on control and experimental livers are shown in Figure 1. As expected, control livers showed cell cords starting from the centrilobular vein, divided by the hepatic sinusoids (Figure 1A). The liver of CM-fed rats showed a significative increase in the number of Kupffer cells (see Supplementary Figure S1 for statistical analysis) and the presence of inflammatory infiltrates (Figure 1B). The DM-fed rat livers showed no differences compared to the control liver (Figure 1C), while an increase in the number of hypertrophic hepatocytes was seen in the livers of HM-fed rats (Figure 1D). The increase in Kupffer cells in CM-fed rats highlighted in the histological evaluation was confirmed by statistical analysis on sections derived from different areas at 40× magnification (Figure S1).

**Figure 2:**
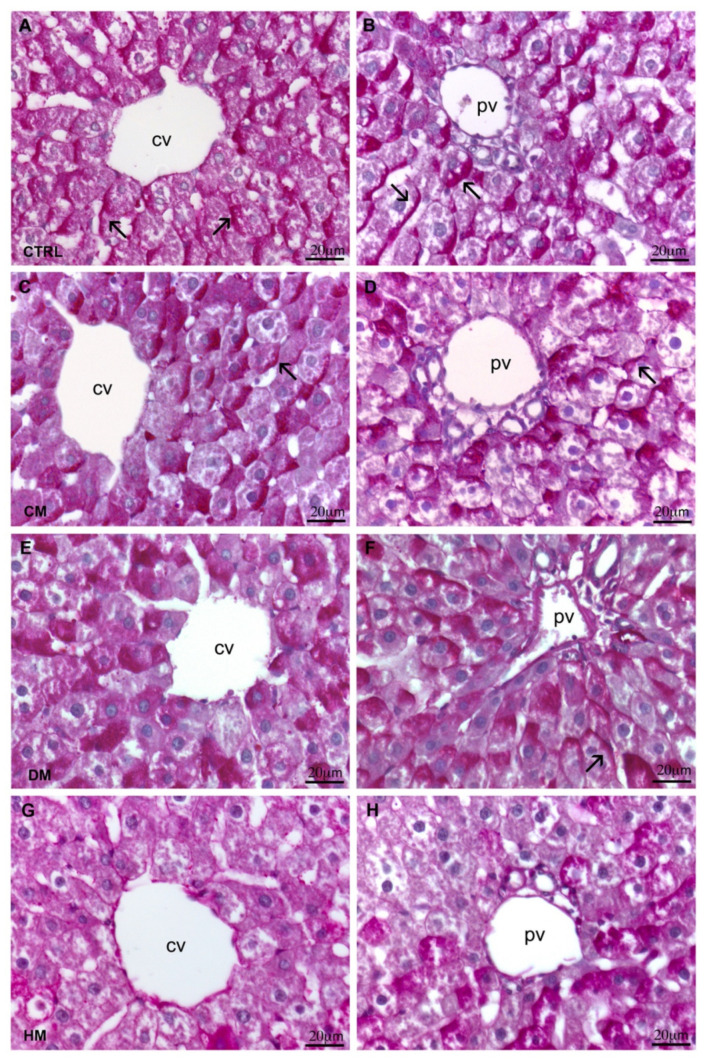
PAS staining: (**A**,**B**) Centrilobular vein and portal triad of control livers display PAS positivity localized at one pole of the hepatocytes (arrows). (**C**,**D**) In CM-fed rat livers, the same PAS positivity disposition of control livers is noticeable in both areas (arrows); (**E**) centrilobular vein of DM-fed rats exhibits widespread PAS positivity within the hepatocytes. (**F**) Portal triad area of DM-fed rats displays positivity localized at one pole of the cells (arrows). (**G**,**H**) In HM-fed rats, a significant decrease in PAS positivity is detectable in both areas. Magnification: 40×, cv: centrilobular vein; pv: portal vein.

In the control, CM- and DM-fed rat livers, PAS-staining using to underline mucin, glycogen and glycoproteins results are distributed both in the centrilobular and in the peripheral zone at the portal triads. In the control (Figure 2A,B) and CM livers (Figure 2C,D), PAS positivity was much stronger at one pole of the hepatocytes surrounding the centrilobular and portal triads. The same pattern was seen at the portal triads area for DM (Figure 2F), while in the centrilobular zone, PAS positivity was instead widespread in the cells (Figure 2E). In the HM group, a clear decrease in PAS reactivity was observed around the centrilobular vein (Figure 2G), while in the area of the portal triad, when present, PAS positivity was diffused within the cytoplasm (Figure 2H).

**Figure 3:**
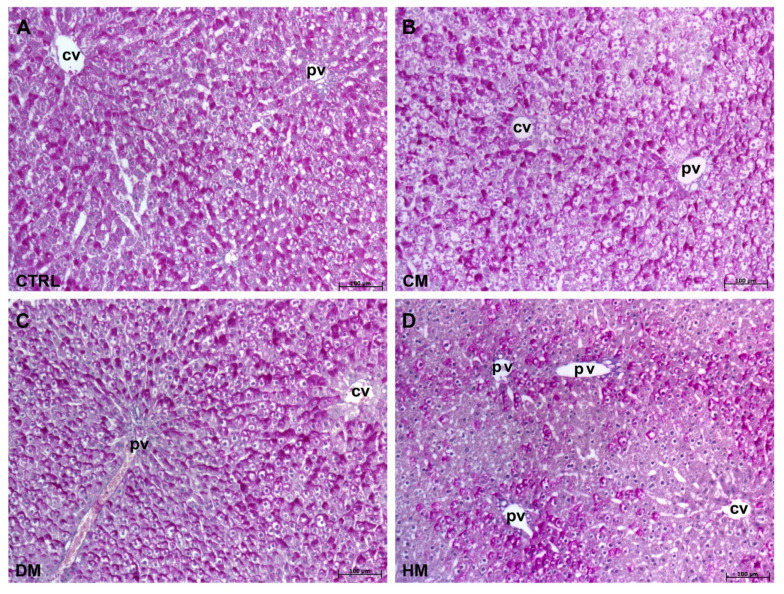
PAS/Dimedone staining: (**A**) Control livers display an equal distribution of positivity in both centrilobular and portal areas. (**B**) CM-fed rat livers display no differences in PAS positivity distribution if compared to control. (**C**) DM-fed rats livers display no differences in PAS positivity distribution if compared to control and to CM-fed rat livers. (**D**) HM-fed rat livers with decreased positivity in both areas, especially around the centrilobular vein. Magnification: 10×. cv: centrilobular vein; pv: portal vein.

The liver sections were stained with PAS/dimedone to highlight the PAS positivity specifically ascribable to glycogen. As already highlighted with standard PAS staining, both pericentrilobular and periportal areas of control (Figure 3A) and CM-fed rat livers (Figure 3B) did not show any visible difference in the glycogen distribution, which was mainly arranged at hepatocyte poles. However, in CM-fed livers, some areas displayed less marked PAS positivity. No differences were found between the glycogen arrangements between DM (Figure 3C) and control liver parenchyma. Also, in the HM-fed group, the standard PAS staining was confirmed, and decreased positivity was observed, especially around the centrilobular vein, mostly distributed within the hepatocytes (Figure 3D).

In addition, the administration of the different milks did not appear to modify the distribution of PAS-positive compounds other than glycogen. The PAS/diastase staining, which provides data relating to all positive PAS substances except glycogen, did not report differences among the different liver specimens.

**Figure 4:**
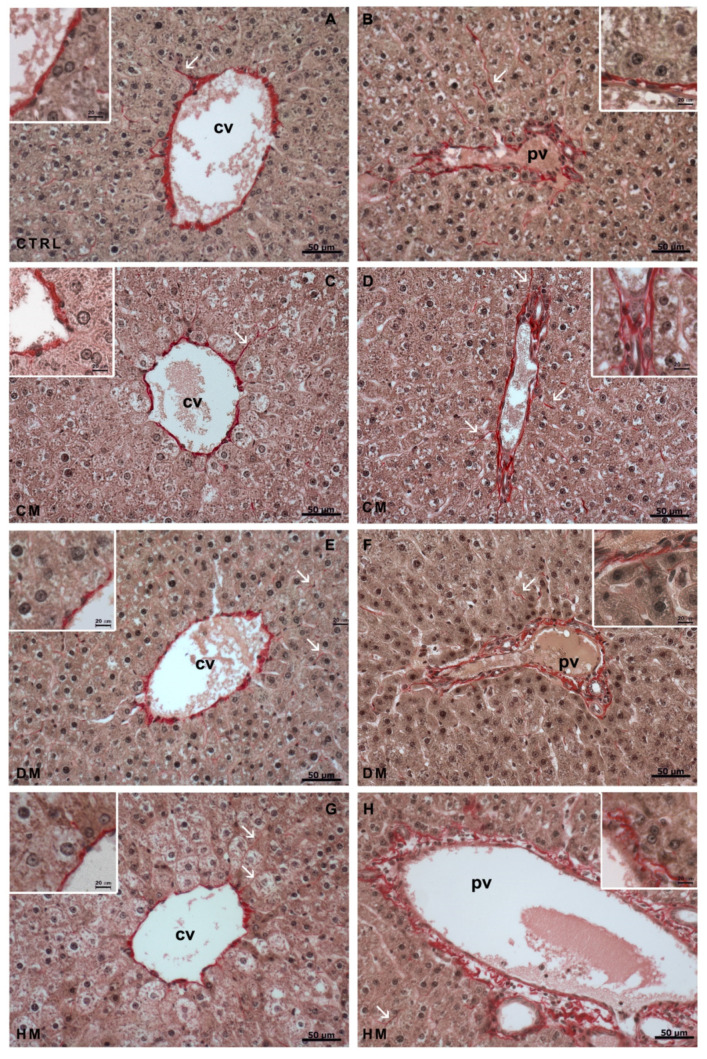
Sirius Red staining: (**A**,**B**) In control livers, a modest layer of connective is detected in both areas and within the space of Disse (arrows); (**C**,**D**) In CM-fed rat livers, a slight increase in connective thickness of both areas is noticeable; (**E**,**F**) In DM-fed rat livers, no differences are detected if compared to control livers; (**G**,**H**) centrilobular vein of HM-fed rat livers displays a slight decrease in thickness, while no differences are reported in the portal triad. Arrows: space of Disse. cv: centrilobular vein; pv: portal vein.

Sirius Red staining was carried out to highlight the presence of connective tissue in the liver parenchyma. A modest layer of connective tissue was seen surrounding the centrilobular vein of the control liver, as well as the vessels and the bile duct of portal triads (Figure 4A,B). Furthermore, moderate presence of connective tissue was observed within the spaces of Disse (arrows). The thickness of the connective tissue layer surrounding both the portal triad and centrilobular vein was slightly increased in CM-fed rat livers (Figure 4D), while the DM-fed rat livers (Figure 4E,F) did not show any particular difference compared to the liver of control rats, both at the centrilobular and the peripheral areas. The amount of connective tissue within the spaces of Disse did not seem to vary either. On the contrary, a slight decrease in the connective tissue thickness around the portal triad of HM-fed rats was observed (Figure 4H).

### 3.2. Glucose Homeostasis

**Figure 5:**
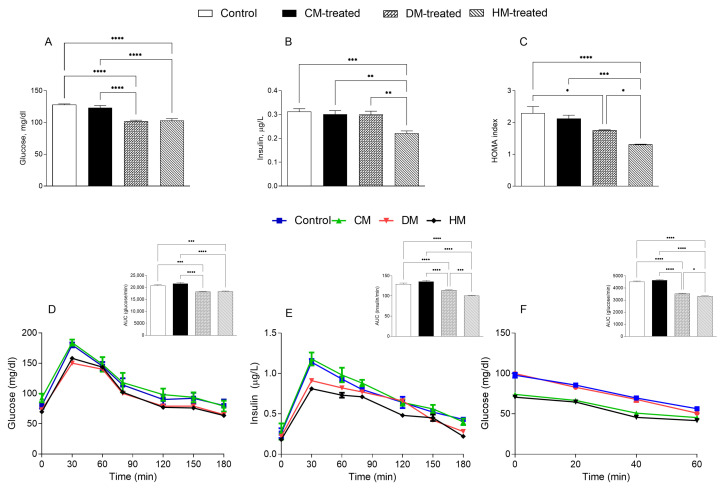
Glucose metabolism parameters: (**A**). Plasma levels of glucose and (**B**) insulin. (**C**) Homa-index. (**D**) Plasma glucose and (**E**) insulin concentrations at different time points after glucose load and (**F**) insulin tolerance test. Histograms in panels (**C**–**E**) represent the area under curve (AUC) related to each condition. Data are presented as means ± SEM from *n* = 7 animals/group. * *p* < 0.05; ** *p* < 0.01; *** *p* < 0.001; **** *p* < 0.0001.

In order to investigate the effects of different milk supplementation on glucose metabolism, different serum parameters were analyzed. In line with our previous results [23], glucose levels were significantly lower in HM- and DM-fed rats compared with control and CM-treated groups, while the lowest levels of insulin were measured in HM-fed rats (Figure 5A,B). Thus, compared with controls, a marked reduction in HOMA index was observed in the DM and HM groups (HM < DM) (Figure 5C). In addition, HM- and DM-treated rats showed higher tolerance to glucose loading than CM-treated and control rats, assessed as the “area under the curve” (AUC) of glucose levels over time (Figure 5D). Consistently, HM- and DM-fed animals showed a significant reduction in insulin levels (AUC) after glucose loading compared with the other groups (HM < DM) (Figure 5E). In addition, upon insulin administration, better glucose reduction (AUC HM < DM) was detected in DM and HM-fed rats compared with CM-treated or control groups (Figure 5F).

### 3.3. Hepatic Inflammatory and Oxidative States

**Figure 6:**
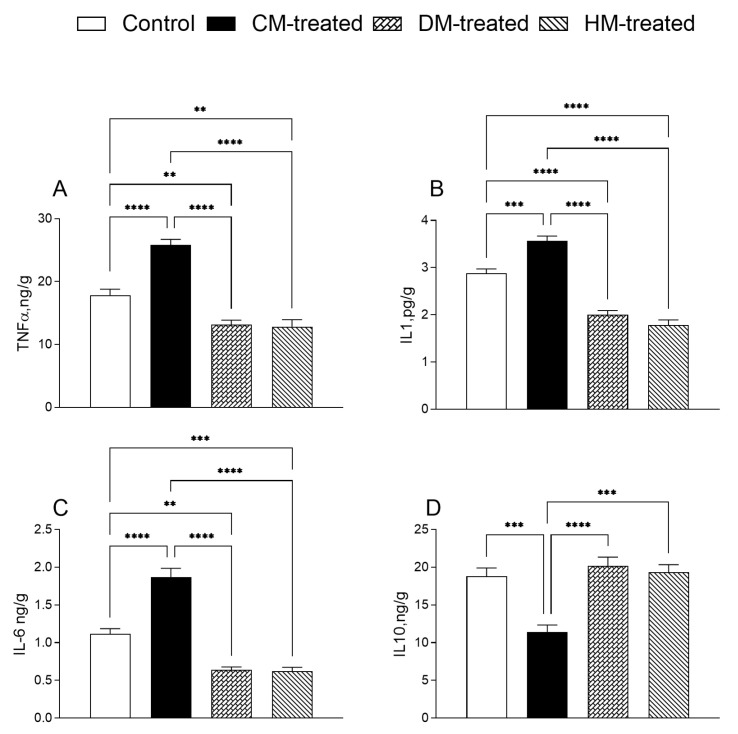
Parameters of the inflammatory state measured in the liver: (**A**) Tumor necrosis factor- α (TNF-α); (**B**) interleukin-1 (IL-1); (**C**) interleukin-6 (IL-6) and (**D**) interleukin-10 (IL-10) levels were measured in the hepatic homogenates in each experimental group. Data are presented as means ± SEM from *n* = 7 animals/group. ** *p* < 0.01; *** *p* < 0.001; **** *p* < 0.0001.

A series of experiments were run to evaluate the effects of milk supplementations on hepatic inflammatory and oxidative states. The inflammatory profile was improved in the DM and HM groups compared with the other two groups. The levels of the proinflammatory cytokines TNF-α, IL-1 and IL-6 were significantly reduced in the DM and HM groups (Figure 6A–C), while the extent of the anti-inflammatory cytokine IL-10 was significantly higher in the DM and HM groups compared with the CM group (Figure 6D).

**Figure 7:**
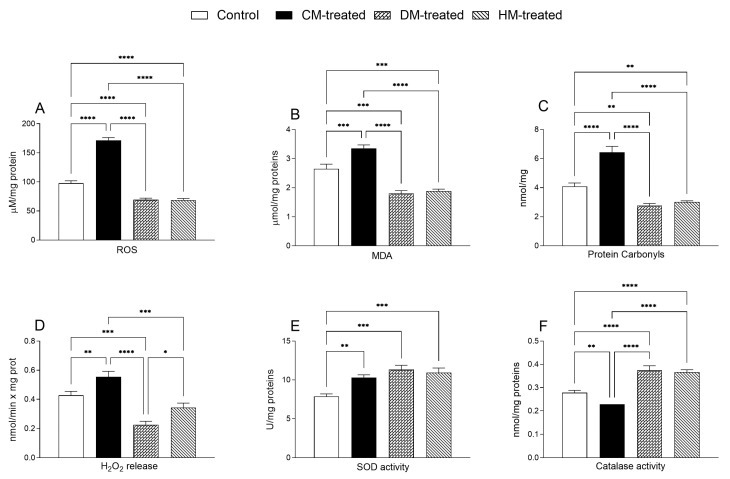
Parameters of the oxidative state measured in the hepatic tissues: (**A**) Reactive oxygen species (ROS), (**B**) malonyldialdeide (MDA) and (**C**) protein carbonyls (PC) were measured in homogenates of hepatic tissue; (**D**) H_2_O_2_ release, (**E**) superoxide dismutase and (**F**) catalase activities were measured in hepatic mitochondrial fraction in each experimental group. Data are presented as means ± SEM from *n* = 7 animals/group. * *p* < 0.05, ** *p* <0.01, *** *p* < 0.001; **** *p* < 0.0001.

In liver homogenates, an amelioration of oxidative state was observed in DM- and HM-treated rats, as demonstrated by the reduced levels of ROS, MDA, PC and H_2_O_2_ release compared with control or CM-fed animals (Figure 7A–D), whereas an increase in mitochondrial SOD activity was observed in all milk-treated groups (Figure 7E). DM- and HM-treated groups also showed increased catalase activity, while a significant reduction was observed in the CM-fed animals (Figure 7F).

### 3.4. Mitochondrial AQP8 Is Significantly Reduced in DM-Fed Rats

**Figure 8:**
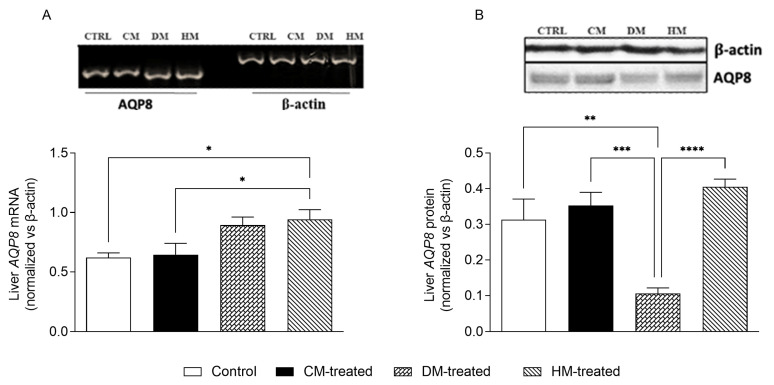
Expression analysis of AQP8: (**A**) At the top is the representative agarose gel showing the expression profiles of the *AQP8* mRNA in different conditions. The levels of b-actin measured in the same samples of total RNA are also shown. At the bottom, the histogram shows the expression levels of *AQP8* normalized against those of β-actin. (**B**) At the top is the representative Western blot showing the AQP8 immunoreactive bands in the 8000× *g* gravitational fraction of liver mitochondria from different experimental conditions. The β-actin protein detected in the whole homogenate of the same liver samples is also shown. At the bottom, the histogram shows the expression levels of mitochondrial AQP8 normalized against those of β-actin in the whole liver homogenates. Data are presented as means ± SEM from five independent preparations. * *p* < 0.05, ** *p* < 0.01, *** *p* < 0.001; **** *p* < 0.0001.

Samples of the livers of CM-, DM- and HM-fed rats were used to evaluate the mRNA and protein expression of AQP8 compared with that of control animals. The AQP8 protein was investigated in the mitochondrial fraction due to its presence and relevance in hepatocyte mitochondrial function [45]. The transcriptional expression of *AQP8* was evaluated by RT-PCR after normalization against the housekeeper gene β-actin. A slight increase in the *AQP8* mRNA level was seen in DM-fed and HM-fed rats when compared with control animals, while the level of the *AQP8* transcript was unchanged in the CM condition (Figure 8A). The liver mitochondrial fraction was used to assess the level of AQP8 protein in this organelle by Western blotting using a polyclonal antibody directed against its N-terminus (see Section 2). As seen in previous works [43], mitochondrial AQP8 was detected as a 28 kDa immunoreactive band (Figure 8B; top), whose expression levels were normalized against those of β-actin. A marked reduction (about −60%) in mitochondrial AQP8 was observed in the liver of DM-fed rats compared with the other groups (Figure 8B; bottom).

### 3.5. Liver AQP9 and Glycerol Permeability Are Increased in Rats Supplemented with Cow Milk

**Figure 9:**
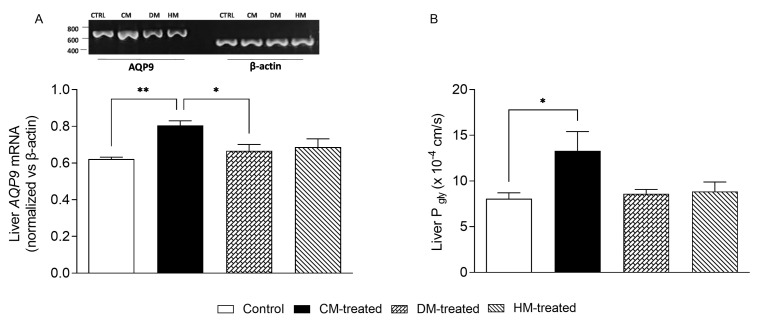
Expression analysis of liver AQP9 and membrane glycerol permeability: (**A**) Representative agarose gel showing the transcriptional expression profiles of *AQP9* in different conditions. The levels of β-actin measured in the same samples of total RNA are also shown. The histogram shows the expression levels of *AQP9* normalized against those of b-actin. (**B**) Glycerol permeability (*P_gly_*) of vesicles of the sinusoidal membrane domain of hepatocytes assessed at 20 °C by stopped-flow light scattering. The *P_gly_* value of CM-fed rats is significantly higher than that of control animals, while the glycerol permeability of the DM and HM vesicles is not different. Each value represents the mean ± SEM from five independent preparations. * *p* < 0.05; ** *p* <0.01.

We then evaluated the gene and protein expression of AQP9, an aquaglyceroporin highly expressed in hepatocytes with important roles in glucose and lipid homeostasis. Samples of the same liver specimens were also used to biophysically measure the membrane permeability to glycerol. By RT-PCR, a significant increase in *AQP9* transcript (+35%) was observed in CM-fed rats compared with control animals, while the mRNA levels of *AQP9* in DM and HM were substantially unchanged (Figure 9A). To investigate whether the increase in *AQP9* expression in the liver of CM-fed rats was accompanied by an increase in hepatocyte glycerol permeability, a series of stopped-flow light scattering experiments were run using vesicles prepared from liver gravitational fractions enriched in sinusoidal membrane, the domain in which AQP9 is localized in hepatocytes. Consistent with the increased expression of AQP9 described above, the glycerol permeability (*P_gly_*; cm/s) of the CM-fed membrane vesicles was significantly higher than that of control rats whose diet was not supplemented by any milk (13.31 ± 2.1 vs. 8.05 ± 0.65 × 10^−4^ cm/s, respectively; Figure 9B). The *P_gly_* values of the DM and HM membrane vesicles were not statistically different from those of the control rats (Figure 9B).

## 4. Discussion

We previously conducted studies using rodent models aimed at comparing the nutritional, immunomodulatory and antioxidant effects of isoenergetic supplementation with human, donkey or cow milk [18,19,23,24,25]. DM and HM intake were found to reduce the accumulation of body lipids by improving the utilization of fat as metabolic fuel in hepatic mitochondria, while in CM-fed animals we found lower energy expenditure, associated with higher levels of body lipids [25]. The different efficacy of various types of milk in modulating lipid storage, utilization and peroxidation, mitochondrial ROS production and oxidative state highlights the functional impact of different milks, both in metabolic and inflammatory homeostasis. However, the framework of the studies leading to the identification of pathways involved and the cross-regulations incorporating all signaling events activated in response to the intake of specific nutrients is far from complete. Aquaporins have important implications in energy metabolism, redox signaling, oxidative stress and mitochondrial ROS release [71].

The liver is the chief organ that coordinates the metabolic flexibility of the organism, balancing immune and metabolic homeostasis [35,72,73]. The liver manages the flow of nutrients and endocrine and immune mediators, delivered via portal blood from gut and visceral adipose tissue, which modulate the hepatic metabolism influencing the bioenergetic regulation in hepatic mitochondria, organelles acting as a metabolic hub for the regulation of hepatocyte homeostasis [32]. In addition, the liver plays key metabolic functions. As the main target of insulin and glucagon signaling, the liver is pivotal in modulating glucose blood levels by regulating glycogen synthesis and gluconeogenesis [74].

In the present work, we found that DM and HM isoenergetic supplementations results in a reduction in hepatic lipid content with a decrease in oxidative stress, hepatic inflammatory markers and collagen and Kupffer cells. However, it should be noted that the group of control rats did not represent a pathological control, because the related animals received a standard diet without any supplementation. These results are in line with the enhanced hepatic mitochondrial function observed in our previous study [25]. We also found that the effects exerted by milk supplementation on rat hepatic lipid and glucose metabolism and inflammatory and redox states involve the main hepatocyte aquaporins, AQP8 and AQP9.

Liver is organized in clearly demarcated metabolic zones. Gluconeogenesis takes place mainly in the periportal zone, while glycolysis takes place in pericentral hepatocytes [75]. Furthermore, initial hepatic glycogen deposition occurs at the plasma membrane, resulting in accumulation at the periphery; then, the synthesized glycogen moves into the cytosol of liver cells [76]. Our histological data are consistent with both the metabolic zonation and the arrangement of glycogen in the hepatocyte. It is noteworthy that hepatic glycogen accumulation increases in DM-fed rats compared with other treatments, whereas its reduction was observed in HM-fed rats in centrilobular zone. In addition, both in donkey- and human-milk-treated groups, a reduction in hepatic lipid accumulation was observed. Donkey milk has a closer composition to human milk than ruminant milk, since human and donkey milk have a higher lactose content (about 7 g/100 g) than cow milk (about 4.6%) [77]. Lactose contains the simple sugar galactose which, unlike the glucose and fructose parts of sucrose, switches completely into liver glycogen upon absorption [78]. This glycogenic effect can have an impact on glucose utilization in the whole body and on insulin sensitivity. Indeed, it was demonstrated that among milk sugars, the galactose moiety of lactose improved hepatic insulin sensitivity in nondiabetic, normal rats [79]. Our observations confirm that the intake of DM and HM, despite their high sugar content, results in improved glucose metabolism, as also indicated by the attenuated glycaemic response, lower insulin levels and reduced HOMA index. These results are in line with other studies [23,80] and confirm the ability of DM and HM to ameliorate glucose homeostasis.

The differences in glycogen and lipid depots among DM- and HM-treated rats could be explained, at least in part, by the hepatic mitochondrial substrate oxidation [81]. We observed a significant increase in the number of hypertrophic cells in the hepatocytes of HM-fed rats, especially in zone 3. Previous studies consider hypertrophy to be a reversible change that helps the organism through increased metabolic capacity [82]. This suggests that according to previous research [25], supplementation with human milk induces an increase in energy metabolism. Indeed, we previously found an increase in liver mitochondrial function in both donkey- and human-treated rats. However, the increase in metabolic activity found in the DM and HM groups is not translated into a worsening of the oxidative state. As matter of fact, here, we observed that the intake of DM and HM results in an increase in enzymatic antioxidant defenses (SOD and CAT activities), accompanied by a significant reduction in ROS, MDA, carbonylated proteins levels and mitochondrial release of H_2_O_2_.

DM and HM intake also positively affect the inflammatory profile. Here, in line with previous results obtained with sera [23], we observed lower concentrations of TNF-α, IL-1 and IL-6 in the liver, whereas only CM-fed animals reduced the hepatic levels of IL-10, an anti-inflammatory marker. In addition, the histological analyses showed an increased number of Kupffer cells and a moderate presence of inflammatory infiltrates, as well as an increase in lipid droplets in rats fed with cow’s milk supplementation. These findings are consistent with other studies that have shown an increase in the number of liver macrophages after being fed a high-fat diet [83]. This finding might suggest a potential proinflammatory effect of such type of milk; however, the measurements of serum parameters of proinflammatory cytokines in rats fed cow’s milk carried out previously [25] never showed a situation of systemic inflammation. Moreover, from our investigations, the moderate inflammatory state found in CM-fed rats is not associated with a clear increase in collagen or Ito cells, which are responsible, when stimulated by inflammatory signals, for collagen production and fibrosis development [84].

The reduced level of mitochondrial AQP8 found in the liver of DM-fed rats is in line with the decrease in hepatic oxidative stress seen in this rat specimen, since one of the main functions ascribed to mitochondrial AQP8 is that of facilitating the release of hydrogen peroxide out of these organelles [85]. The reduction in AQP8 protein levels in the liver mitochondria of DM-fed rats is most likely performed at the post-translational level, since the increase seen at the mRNA is low. In a previous work using yeast to study the targeting of AQP8 in mitochondria, it was found that this AQP was undergoing hydrolytic removal of a short peptide at the N-terminal end before entering the organelle without affecting the molecular permeability of the channel. Reduced efflux of H_2_O_2_ generated during oxidative stress in the pancreatic β-cell mitochondria was ascribed to the loss of mitochondrial AQP8 [86,87]. In the same work, AQP8 was also seen to increase after cytokine exposure, both in vitro and in vivo. This upregulation was suggested to occur through the NF-κB consensus sequence present in the AQP8 gene promoter. This is consistent with the reduction in hepatic inflammatory markers whose production involves the NF-κB signaling pathway observed in the present study. The mechanism by which donkey milk supplementation but not cow or human milk administration reduces AQP8 targeting in hepatocyte mitochondria remains to be defined, being a matter for future studies.

The increase in hepatocyte AQP9 seen in CM-fed rats fits well with the higher hepatic fat content and glycerol permeability induced by cow milk. AQP9 is the main, if not the only, facilitated route through which glycerol is imported by hepatocytes [50,55]. After its import within the cell, glycerol is phosphorylated to G3P which, depending on the nutritional status, can enter the gluconeogenetic pathway during fasting or be used in the synthesis of TAGs when fat must be accumulated in the cell. It is therefore reasonable to think that the accumulation of lipids induced by cow milk supplementation also involves an increase in AQP9 expression to raise the hepatic uptake of glycerol. The involvement of AQP9 in rodent hepatic lipid metabolism is sex-specific [59]. In a recent study, only male *Aqp9* knockout mice showed a reduced hepatic secretion of TAGs and an elevated expression of peroxisome proliferator-activated receptor α, which was suggested to promote an increased hepatic fatty acid oxidation [81]. This may help understand the mechanism through which cow milk upregulates liver AQP9. Further studies are being planned with the aim of resolving some of the critical issues in this design. It is being hypothesized to use AQP8 or AQP9 KO mice in order to better detail the functional involvement of AQP8 or 9 in mediating the effects of milk from different sources. Moreover, we are aware of the species-specific differences that result in response to the intake of milk from different species. These effects are reflected in growth, metabolic, immunological and neurological programming but are significantly crucial in infants fed exclusively milk. To explore this further, we have already planned a new study where different kinds of milk will be administered to newborn rats.

## 5. Conclusions

In conclusion, compared with rats with no milk supplementation, DM- and HM-fed rats had reduced hepatic lipid content with enhanced mitochondrial function and decreased oxidative stress. However, while DM-fed rats showed a marked reduction in AQP8, a H_2_O_2_ channel, in their hepatic mitochondria, the level of mitochondrial AQP8 in the liver of HM-fed animals was similar to that of CM-fed and control rats. The beneficial effect of DM and HM was also accompanied by a reduction in hepatic inflammatory markers and the presence of Kupffer cells. CM-fed rats showed higher hepatic fat content and increased AQP9 and glycerol permeability.

## Figures and Tables

**Table 1:** Diet composition.

Diet Composition	Chow	CM	DM	HM	Chow + CM	Chow + DM	Chow + HM
Protein (%)	29	30.7	18	12.5	29.2	27.5	26.7
Lipid (%)	10.6	34	4.7	35	13.9	9.8	14.1
Carbohydrate (%)	60	35.5	77.3	52.5	56.5	62.4	58.9
Energy density (kJ/g)	15.8	3.46	1.48	3.24	14.0	13.8	14.0

**Table 2:** Dietary energy intake.

Energy Intake	Control	CM-Treated	DM-Treated	HM-Treated
Total Energy (kJ)	12,432 ± 176	14,071 ± 204 *	14,288 ± 222 *	14,095 ± 202 *
Chow (kJ)	12,432 ± 164	12,064 ± 184 (85.74%)	12,280 ± 203 (85.95%)	12,091 ± 188 (85.78%)
Milk (kJ)		2007 ± 14 (14.26%)	2008 ± 18 (14.05%)	2004 ± 14 (14.22%)

* *p* < 0.05 vs. control group.

## Data Availability

The data presented in this study are presented in the results section and are available on request from the corresponding author.

## Supplementary Materials

The following supporting information can be downloaded at: https://www.mdpi.com/article/10.3390/nu15163651/s1, Table S1: Body weight gain in differently treated rats; Figure S1: Statistical analyses of Kupffer cells on sections derived from different areas at 40× magnification.
