# Supplementary material for: Hepatocyte Aquaporins AQP8 and AQP9 Are Engaged in the Hepatic Lipid and Glucose Metabolism Modulating the Inflammatory and Redox State in Milk-Supplemented Rats
Source: Nutrients. 2023 Aug 20;15(16):3651. doi: 10.3390/nu15163651 (PMC10459073; doi:10.3390/nu15163651)

Table S1. Body weight gain in differently treated rats

	Control	CM	DM	HM
Initial body weight (g)	327±3.0	354±4	362±3.9	340±4.7
Final body weight (g)	469±2.5	529±4.8*	503±4	478±4.4
Body weight gain (g)	142±2.5	175±2.25*	141±1.3	138±0.9

$p < 0.05$ vs control, DM- and HM- fed rats

Figure S1. Statistical analyses of Kupffer cells on sections derived from different areas at 40x magnification. Data are presented as means ± SEM. **** $p < 0.0001$.

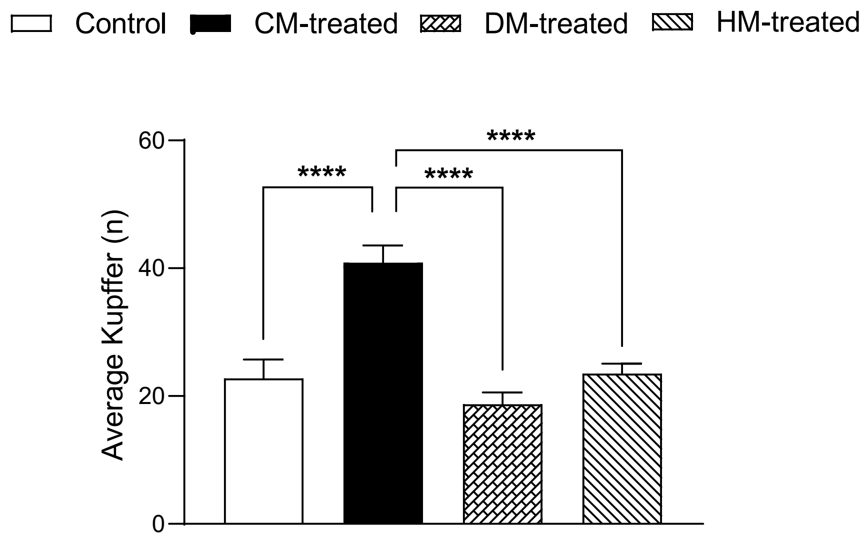

Supplement: Supplementary file 1 [file nutrients-15-03651-s001.zip › nutrients-2515455-supplementary.pdf]
